# Serum Leucine-Rich Alpha-2 Glycoprotein 1 Levels in Patients with Lipodystrophy Syndromes

**DOI:** 10.3390/biom14111474

**Published:** 2024-11-19

**Authors:** Michelle Krienke, Susan Kralisch, Leonie Wagner, Anke Tönjes, Konstanze Miehle

**Affiliations:** Medical Department III—Endocrinology, Nephrology, Rheumatology, University of Leipzig Medical Center, Liebigstraße 20, 04103 Leipzig, Germanysusan.kralisch@medizin.uni-leipzig.de (S.K.); leonie.wagner@medizin.uni-leipzig.de (L.W.); anke.toenjes@medizin.uni-leipzig.de (A.T.)

**Keywords:** adipokine, diabetes mellitus, insulin resistance, leucine-rich 2-glycoprotein 1, lipodystrophy, obesity

## Abstract

Serum concentrations of leucine-rich alpha-2 glycoprotein 1 (LRG1) are elevated in several cardio-metabolic and inflammatory diseases. LRG1 also plays an important role in the development of hepatic steatosis and insulin resistance. In lipodystrophies (LDs), severe cardio-metabolic complications can be observed. The dysregulation of several adipokines plays a significant role in the clinical manifestation of this syndrome. To date, there have been no studies of LRG1 levels in non-HIV-LD patients. We performed a cross-sectional analysis of LRG1 serum levels in 60 patients with non-HIV-associated LD and in 60 age-, sex-, and BMI-matched healthy controls. Furthermore, we investigated the gene expression of *Lrg1* in a *mouse* model of generalised LD. No significant difference was found in the median concentration of LRG1 serum levels between LD patients (18.2 ng/L; interquartile range 8.3 ng/L) and healthy controls (17.8 ng/L; interquartile range 11.0 ng/L). LRG1 serum concentrations correlated positively with CRP serum levels (*p* < 0.001). *Lrg1* mRNA expression was downregulated in the adipose tissue, whereas in the liver, no difference in *Lrg1* expression between LD and wild-type *mice* was detected. In summary, circulating levels of LRG1 are associated with low-grade inflammation but cannot distinguish between patients with LD and controls.

## 1. Introduction

Leucine-rich alpha-2 glycoprotein 1 (LRG1) is a protein that is part of the leucine-rich repeat (LRR) family of proteins. It is involved in various biological processes such as inflammation, immune response, and cell adhesion [1]. Under physiological conditions, LRG1 is secreted primarily by the liver and has been found to be upregulated in various diseases such as cancer, cardiovascular disease, and inflammatory conditions [1]. Since recent studies revealed that LRG1 is also secreted by adipocytes, it has been studied in the context of adipose tissue and obesity [2]. LRG1 expression is upregulated in adipose tissue in obese individuals and may be involved in the modulation of adipogenesis, inflammation, and insulin resistance in adipose tissue [3].

In lipodystrophy (LD) syndromes, a group of rare acquired or congenital disorders, cardio-metabolic complications similar to those found in obesity can be found. Due to loss of adipose tissue in either a general or partial way (which is the pathognomonic symptom of LD), the capacity of adipocyte tissue depots for storage of surplus energy is precociously exceeded in LD. This leads to an increase in circulating lipids and ectopic lipid accumulation with concomitant organ dysfunction [4]. As in obesity, an adverse adipokine secretion profile has been described for LD. Serum concentrations of leptin and adiponectin are reduced in LD [5]. These two adipokines are crucial for an adequate control of energy homeostasis and glucose metabolism [6]. Substitution with recombinant leptin was described as a specific treatment for metabolic complications in LD already 20 years ago [7]. However, individual response to treatment is highly variable, especially in partial LD, which is the most common LD subgroup in Europe [8,9]. Hence, there is a need for finding sufficient predictors for individual responses to leptin substitution as well as for new treatment options in LD.

To the best of our knowledge, LRG1 serum levels have not been investigated in patients with LD so far. We performed a cross-sectional analysis of LRG1 serum levels in non-HIV LD patients and a sex-, age-, and BMI-matched healthy control group. We hypothesised that LRG1 is upregulated in patients with LD and may contribute to vascular and metabolic consequences.

## 2. Materials and Methods

### 2.1. Patients

A detailed description of the patient and control groups has been given recently [10]. In short, 60 patients (48 females/12 males) with generalised (*n* = 5) and partial (*n* = 55) LD and 60 healthy, age-, BMI-, and sex-matched non-LD subjects were included in our study. Age ranged from 16 to 75 years, and the BMI ranged from 17.4 to 46.1 kg/m^2^. LD was diagnosed according to the 2016’s Multi-Society Practice Guideline [11]. Hepatic steatosis was assessed in the LD cohort only using vibration-controlled transient elastography (FibroScan; Echosens, Paris, France) with measurement of controlled attenuation parameter (CAP) using either M or XL probe. CAP > 288 dB/m was used as a cut-off for the detection of steatosis [12].

Written informed consent was obtained from all participants prior to enrolment. This study was approved by the local Ethics Committee of the University of Leipzig.

### 2.2. Laboratory Assays

Serum adipokine concentrations were determined using enzyme-linked immunosorbent assays from Mediagnost, Reutlingen, Germany (leptin and total adiponectin measurement) and Abcam, Cambridge, United Kingdom (LRG1 measurement), according to the manufacturer’s instructions. Measurements of the metabolic and inflammatory parameters, i.e., fasting insulin (FI), fasting glucose (FG), glycated haemoglobin A1c (HbA1c), creatinine, triglycerides (TGs), cholesterol, high-density lipoprotein (HDL) cholesterol, low-density lipoprotein (LDL) cholesterol, free fatty acids (FFAs), and CRP, were performed by standardised laboratory methods in the Institute of Laboratory Medicine, Clinical Chemistry, and Molecular Diagnostics, University of Leipzig Medical Centre. Blood samples were collected after an overnight fast. The homeostasis model assessment of insulin resistance (HOMA-IR) was calculated using the method described by Matthews et al. [13], and the CKD-EPI equation [14] was used to calculate the estimated glomerular filtration rate (eGFR).

### 2.3. Mouse Experiments

Animal experiments were performed in accordance with the guidelines approved by the State of Saxony, Germany, and in compliance with the recommendations of the local animal Ethics Committee (TVV39/21). In this study, we used male transgenic aP2-SREBP1c *mice* (SREBP1c-Tg, *n* = 5) on a C57Bl/6 background to study the congenital generalised LD phenotype [15]. Male wild-type SREBP1c *mice* (SREBP1c-WT, *n* = 5), also on a C57Bl/6 background, served as controls. All *mice* were maintained at 23 °C on a 12 h light/dark cycle, fed standard chow (Ssniff, Soest, Germany), and given ad libitum access to water and food. *Mice* were sacrificed at an age of 15 weeks, and tissues (inguinal white adipose tissue (iWAT), brown adipose tissue (BAT), and liver) were collected and snap-frozen for further analysis.

### 2.4. RNA Isolation and Gene Expression Analysis

Isolation of RNA from tissues (iWAT, BAT, and liver) was performed by using the RNeasy Lipid Tissue Mini Kit (Qiagen, Venlo, The Netherlands) according to the manufacturer’s instructions. Subsequently, 1 µg of isolated RNA was transcribed into cDNA by using random hexamer primers and M-MLV reverse transcriptase by Promega (Fitchburg, WI, USA). Quantitative real-time PCR was performed by using the LightCycler-DNA Master SYBR Green I Kit (Roche, Basle, Switzerland) according to standard protocols. *Lrg1* (forward sequence: CCATGTCAGTGTGCA; reverse sequence: AAGAGTGAGAGGTGG) gene expression was calculated using the delta-delta CT method with either *Rplp0* (forward sequence: TCGGGTCCTAGACCAGTGTTC; reverse sequence: AGATTCGGGATATGCTGTTGGC) or *18s* (forward sequence: GTAACCCGTTGAACCCCATT; reverse sequence: CCATCCAATCGGTAGTAGCG) as a reference gene and was adjusted to the control group (SREBP1c-WT).

### 2.5. Statistical Analysis

Statistical analysis of *human* data was performed with SPSS Statistics Version 29 (IBM, Armonk, NY, USA). Data were reported as median and interquartile range. The non-parametric Mann–Whitney U test was used to identify differences between the two groups (LD patients and control group). Correlations were presented using Spearman’s rank correlation method. Results with a *p*-value < 0.05 were considered significant. Statistical analysis of *mouse* data was performed using GraphPad Prism 10 software (GraphPad, Boston, MA, USA). Group comparisons were evaluated using a two-tailed Student’s *t*-test, and a *p*-value of <0.05 was considered statistically significant in all analyses.

## 3. Results

### 3.1. Descriptive Statistics

The clinical and laboratory characteristics of LD patients and controls are shown in Table 1. The median LRG1 concentrations did not show a significant difference between LD patients (18.2 ng/L; interquartile range 8.3 ng/L) and controls (17.8 ng/L; interquartile range 11.0 ng/L) (Table 1). The LD cohort showed significantly higher WHR and SBP as well as a significant malfunction of glucose metabolism (i.e., higher HbA1c, FI, and HOMA-IR) and lipid parameters (i.e., lower HDL cholesterol, LDL cholesterol, and higher TG) compared to the controls. Moreover, serum levels of adiponectin and leptin were significantly lower in LD patients as compared to the healthy controls. There was also a significant difference in the liver enzymes, i.e., higher ASAT, ALAT, and GGT, in LD patients. Comparing LD patients with and without complications, we did not find significant differences in LRG1 serum levels between LD patients with insulin resistance (defined as HOMA-IR ≥ 2.5; *n* = 46) vs. LD patients without insulin resistance (HOMA-IR < 2.5; *n* = 14; *p* = 0.327), LD patients with overt diabetes mellitus (*n* = 38) vs. non-diabetic LD patients (*n* = 22; *p* = 0.709), and LD patients with hepatic steatosis (*n* = 28) vs. no steatosis (*n* = 20; *p* = 0.975). Patients with generalised LD had significantly lower BMI, HDL cholesterol, LDL cholesterol, creatinine, adiponectin levels, and leptin levels than patients with partial LD. In contrast, TGs, eGFR, CRP, ALAT, and ASAT were significantly higher in generalised LD as compared to partial LD. Interestingly, there were no significant differences in LRG1 serum concentration and HbA1c between the two LD subgroups (Supplementary Table S1).

### 3.2. Lrg1 mRNA Expression in Several Tissues of an LD Mouse Model

**Figure 1:**
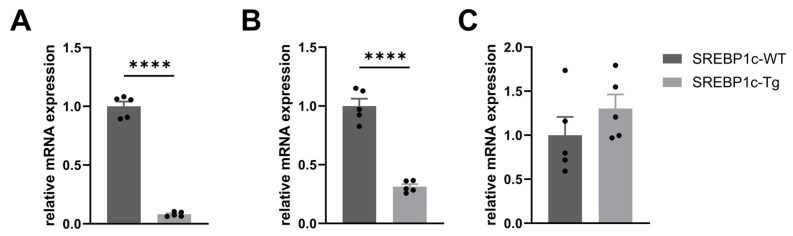
(**A**,**B**) The expression of *Lrg1* in the iWAT and BAT of lipodystrophic SREBP1c-Tg *mice* (*n* = 5) was found to be significantly reduced in comparison to wild-type controls (*n* = 5). (**C**) Hepatic *Lrg1* gene expression in SREBP1c-Tg *mice* exhibited a tendency towards upregulation. Data are presented as means ± SEM. **** *p* ≤ 0.0001, compared to wild-type controls, unpaired *t*-test.

To detect possible differences in *Lrg1* expression between LD *mice* (SREBP1c-Tg) and WT controls (SREBP1c-WT), we performed mRNA analyses in several adipose tissues and livers of both animal cohorts. *Lrg1* mRNA expression was significantly downregulated in the iWAT (*p* < 0.0001) and BAT (*p* < 0.0001) of SREBP1c-Tg *mice* as compared to SREBP1c-WT controls (Figure 1A,B). In contrast, there was a tendency for increased expression of *Lrg1* in the livers of the lipodystrophic SREBP1c-Tg *mice*; however, the observed difference did not achieve statistical significance (Figure 1C).

### 3.3. Correlation Analysis

The entire study cohort was included to test for possible correlations between LRG1 and anthropometric and metabolic parameters. LRG1 serum concentrations correlated positively with CRP serum levels (*p* < 0.001) (Table 2). No significant association was found between LRG1 levels and BMI, WHR, blood pressure, HbA1c, FG, FI, HOMA-IR, cholesterol, HDL cholesterol, LDL cholesterol, TGs, FFAs, creatinine, eGFR, adiponectin, leptin, and liver enzymes (Table 2).

## 4. Discussion

We have investigated serum concentrations of the adipokine LRG1 in a cohort of 60 patients with different subtypes of non-HIV LD. Several studies show that elevated levels of LRG1 are associated with a wide range of diseases, including cancer, heart failure, and inflammatory diseases [16]. High concentrations of LRG1 are closely linked to obesity and might serve as an early obesity marker in overweight adolescents [3]. It appears to be a marker of cardiovascular and all-cause mortality in patients with diabetes mellitus type 2 (T2DM) [17]. He et al. identified LRG1 as a key molecule mediating the crosstalk between adipocytes and hepatocytes in diet-induced hepatosteatosis and insulin resistance by upregulating de novo lipogenesis and downregulating fatty acid beta-oxidation, most likely via sterol regulatory element-binding transcription factor 1 [2]. Although LD is often associated with hepatic steatosis and insulin resistance, we could not find a significant difference in the serum levels of LRG1 between LD patients and the age-, BMI-, and sex-matched healthy controls. Moreover, comparing subgroups of LD patients with vs. without insulin resistance/diabetes mellitus, with vs. without hepatic steatosis, and with generalised LD vs. partial LD does not show any significant differences in LRG1 serum levels.

Independently of concomitant diabetes mellitus, LD itself is associated with a pro-inflammatory state [18]. Compared to the control group, we detect increased CRP as a circulating key inflammatory marker in the serum of our LD cohort. Since LRG1 serum levels do not significantly differ between patients with LD and controls, circulating LRG1 does not seem to have substantial relevance in this inflammatory process. Therefore, it might be supposed that inflammation in LD is due to the increased serum levels of other pro-inflammatory cytokines, e.g., chemerin, progranulin, tumour necrosis factor alpha, and interleukin 6, which have been determined to be significantly elevated in serum of patients with LD as compared to healthy individuals [19,20,21,22].

LRG1 is predominantly secreted by hepatocytes and immune cells [23]. He and co-workers identify adipose tissue as a source of LRG1 protein expression [2] and show significantly higher LRG1 protein levels in adipose tissue than in the liver. Interestingly, and in contrast to other adipokines such as adiponectin and leptin, the circulating levels of LRG1 are not decreased in our patients despite the lack of adipose tissue in LD. Since serum levels reflect the sum of LRG1 secreted by various tissues, it might be speculated that tissue-specific alterations of *Lrg1* mRNA expression and secretion occur in LD. While there are no significant differences in the circulating levels of LRG1 between LD patients and controls, we observe clear tissue-specific expression differences between LD *mice* and the corresponding wild-type controls. Thus, *Lrg1* expression is significantly reduced in the iWAT and BAT of SREBP1c-Tg LD *mice* in comparison to wild-type control animals. In contrast, we identify no significant differences in *Lrg1* mRNA expression in the liver of SREBP1c-Tg *mice* as compared to SREBP1c-WT controls. Rather, there is a trend towards increased expression of *Lrg1* in the liver of LD *mice*, albeit without statistical significance. In diet-induced obese *mice*, *Lrg1* expression correlates with adipogenesis. Moreover, they show significant induction of *Lrg1* mRNA in iWAT [24]. The reduced *Lrg1* expression in our transgenic LD *mice* may reflect adipose tissue dysfunction, which is one of the hallmarks of LD syndromes. Recent studies by Choi et al. indicate that *Lrg1* overexpression is associated with enhanced glucose homeostasis and a reduction in macrophage infiltration in white adipose tissue in an obese *mouse* model [24]. The preserved hepatic *Lrg1* expression of LD *mice* may represent a compensatory mechanism to counterbalance the reduced expression in the dysfunctional adipose tissue. Additionally, Choi et al. identify a reduction in inflammation in the fatty liver of *mice* overexpressing *Lrg1*, indicating a potential anti-inflammatory function of LRG1 in hepatic tissue [24]. Consequently, the amount of hepatic *Lrg1* expression in SREBP1c-Tg *mice* may represent an adaptive response in order to promote anti-inflammatory effects and to preserve liver function in LD. However, the potential role of LRG1 in regulating macrophage infiltration and tissue homeostasis in lipodystrophic *mice* requires further investigation. It would be of interest whether the different mRNA expression profiles in several tissues lead to differences in LRG1 serum concentrations between SREBP1c-Tg *mice* and SREBP1c-WT controls.

Correlating LRG1 levels of the entire study group with anthropometric and metabolic parameters reveals a positive association between LRG1 and CRP, which is consistent with other studies and reflects the pro-inflammatory role of LRG1. Alhammad et al. show a positive correlation of LRG1 serum concentration with CRP and other obesity markers in a group of overweight and obese adolescents [3]. Recent studies have also shown its association with inflammatory bowel disease and suggest LRG1 as a useful biomarker of endoscopic disease activity during treatment [25,26]. LRG1 has also been characterised as an important player in pathological angiogenesis and vascular endothelial dysfunction. In this context, it is considered an enhancer of transforming growth factor beta-induced renal fibrosis in T2DM patients and a predictor of albuminuria and chronic kidney disease progression [27]. In addition, higher levels of LRG1 seem to be generally associated with renal function decline in T2DM patients and may serve as a predictor for the risk of kidney disease progression [28,29]. Interestingly, we could not find an association between serum LRG1 concentration and renal function parameters (e.g., creatinine, eGFR) in our study group. However, we did not measure urinary albumin excretion.

A shortcoming of our study is the small sample size and the heterogeneity of the LD group. We show that circulating LRG1 is no marker for inflammation and metabolic complications in this aetiologically mixed LD cohort even after subdividing the group into generalised and partial LD. However, the terms generalised and partial LD only describe the extent of adipose tissue loss. Within these two groups, there is a huge variability with regard to aetiology, grade of inflammation, as well as the type and severity of metabolic complications [30,31,32]. Moreover, each LD subtype has specific and varying non-metabolic comorbidities, e.g., cardiovascular signs, glomerulonephritis, neurological manifestations, skeletal manifestations, skin anomalies, and premature ageing [11,33,34]. Thus, whether there is an alteration in LGR1 serum levels in some defined LD subtypes cannot be excluded to date.

## 5. Conclusions

In conclusion, we show that LRG1 serum levels in LD patients are not significantly different from a group of age-, sex-, and BMI-matched healthy controls. This indicates that, in contrast to other metabolic and inflammatory diseases, circulating LRG1 is not a main marker of inflammation and insulin resistance in patients with LD. Our study also reveals tissue-specific alterations of *Lrg1* mRNA expression in LD *mice*, with significantly reduced levels in several adipose tissues of SREBP1c-Tg animals as compared to wild-type controls. However, mRNA expression in the liver is comparable between both groups and shows a trend towards increased expression in the liver of LD *mice*. This suggests a complex role for LRG1 in modulating metabolic and inflammatory processes in different tissues.

## Figures and Tables

**Table 1:** Baseline characteristics of the entire study population (*n* = 60 controls and *n* = 60 LD). ALAT, alanine aminotransferase; ASAT, aspartate aminotransferase; BMI, Body mass index; CRP, C-reactive protein; DBP, Diastolic blood pressure; eGFR, estimated glomerular filtration rate; FFAs, free fatty acids; FG, fasting glucose; FI, fasting insulin; GGT, Gamma-glutamyltransferase; HbA1c, Glycosylated haemoglobin A1c; HDL, high-density lipoprotein; LRG1, leucine-rich alpha-2 glycoprotein 1; HOMA-IR, homeostasis model assessment of insulin resistance; LD, lipodystrophy; LDL, low-density lipoprotein; SBP, Systolic blood pressure; TGs, triglycerides; WHR, Waist–hip ratio. Values for median (interquartile range) are shown. * indicates *p* < 0.05 as assessed by Mann–Whitney U test.

	Normal Range	Controls	LD	*p*
*n*		60	60	
LRG1 (ng/L)		18.2 (8.3)	17.6 (10.8)	0.799
Age (years)		39 (22)	42 (24)	0.591
Gender (male/female)		12/48	12/48	-
BMI (kg/m^2^)	18.5–24.9	24.6 (4.9)	25.2 (4.6)	0.193
WHR	F: <0.85; M: <0.90	0.81 (0.11)	0.97 (0.11)	<0.001 *
SBP (mmHg)	<140	122 (22)	131 (19)	<0.001 *
DBP (mmHg)	<90	78 (15)	81 (14)	0.183
HbA1c (%)	<6.5%	5.2 (0.6)	6.0 (2.1)	<0.001 *
HbA1c (mmol/mol)	<48	33.3 (6.3)	42.4 (23.0)	<0.001 *
FG (mmol/L)	3.9–5.6	5.2 (0.8)	5.6 (3.8)	0.020 *
FI (pmol/L)	20–144	51.8 (45.8)	114.9 (113.6)	<0.001 *
HOMA-IR	<2.0	1.7 (1.7)	4.9 (5.8)	<0.001 *
Cholesterol (mmol/L)	<5.20	5.36 (1.35)	5.29 (2.05)	0.258
HDL cholesterol (mmol/L)	>1.03	1.54 (0.59)	0.85 (0.52)	<0.001 *
LDL cholesterol (mmol/L)		3.56 (1.39)	2.74 (1.76)	<0.001 *
TGs (mmol/L)	<1.70	0.98 (0.60)	2.92 (5.82)	<0.001 *
FFAs (mmol/L)	0.10–0.45	0.44 (0.21)	0.61 (0.28)	0.002 *
Creatinine (µmol/L)	45–84	76 (20)	67 (21)	0.011 *
eGFR (ml/min/1.73m^2^)	>90	94.0 (19.0)	100.2 (31.7)	0.043 *
CRP (mg/L)	<5	0.7 (1.5)	1.7 (2.5)	0.016 *
Adiponectin (mg/L)		9.3 (7.7)	2.7 (3.7)	<0.001 *
Leptin (µg/L)		12.0 (13.9)	4.3 (4.7)	<0.001 *
ALAT (µkat/L)	0.17–0.58	0.34 (0.20)	0.49 (0.42)	<0.001 *
ASAT (µkat/L)	0.17–0.6	0.33 (0.08)	0.48 (0.28)	<0.001 *
GGT (µkat/L)	0.1–0.7	0.26 (0.17)	0.65 (0.60)	<0.001 *

**Table 2:** Univariate correlations with LRG1 in the entire study population. r- and *p*-values are given. Abbreviations are indicated in Table 1. * indicates significant correlation as assessed by Spearman’s correlation method.

	Univariate Correlations
	r/*p*
Age (years)	0.147/0.109
Group (LD vs. Non-LD)	-
Gender	-
BMI (kg/m^2^)	−0.015/0.868
WHR	0.059/0.527
SBP (mmHg)	0.074/0.423
DBP (mmHg)	−0.008/0.933
HbA1c (%)	0.053/0.575
HbA1c (mmol/mol)	0.051/0.589
FG (mmol/L)	−0.068/0.462
FI (pmol/L)	0.027//0.771
HOMA-IR	−0.001/0.994
Cholesterol (mmol/L)	−0.071/0.438
HDL cholesterol (mmol/L)	−0.050/0.589
LDL cholesterol (mmol/L)	0.060/0.518
TGs (mmol/L)	−0.018/0.848
FFAs (mmol/L)	−0.140/0.135
Creatinine (µmol/L)	−0.041/0.657
eGFR (ml/min/1.73 m^2^)	−0.049/0.593
CRP (mg/L)	0.455/<0.001 *
Adiponectin (mg/L)	−0.035/0.707
Leptin (µg/L)	0.052/0.575
ALAT (µkat/L)	0.071/0.462
ASAT (µkat/L)	0.104/0.281
GGT (µkat/L)	−0.010/0.916

## Data Availability

The datasets presented in this article are not readily available because the data are part of an ongoing study. Requests to access the datasets should be directed to the corresponding author.

## Supplementary Materials

The following supporting information can be downloaded at: https://www.mdpi.com/article/10.3390/biom14111474/s1, Table S1: Baseline characteristics of the LD population (*n* = 60), partial LD vs. generalized LD.
