# Supplementary material for: Serum Leucine-Rich Alpha-2 Glycoprotein 1 Levels in Patients with Lipodystrophy Syndromes
Source: Biomolecules. 2024 Nov 19;14(11):1474. doi: 10.3390/biom14111474 (PMC11592172; doi:10.3390/biom14111474)
Supplement: Supplementary file 1 [file biomolecules-14-01474-s001.zip › biomolecules-3205343-supplementary.pdf]

Supplementary Materials

Table S1. Baseline characteristics of the LD population ($n = 60$), partial LD vs. generalized LD. ALAT, alanine aminotransferase; ASAT, aspartate aminotransferase; BMI, Body mass-index; CRP, C reactive protein; DBP, Diastolic blood pressure; eGFR, Estimated glomerular filtration rate; FFA, Free fatty acids; FG, Fasting glucose; FI, Fasting insulin; GGT, Gamma-glutamyltransferase; HbA1c, Glycosylated hemoglobin A1c; HDL, High-density lipoprotein; LRG1, leucine-rich alpha-2 glycoprotein 1; HOMA-IR, Homeostasis model assessment of insulin resistance; LD, Lipodystrophy; LDL, Low-density lipoprotein; SBP, Systolic blood pressure; TG, Triglycerides; WHR, Waist-hip-ratio. Values for median (interquartile range) are shown. * indicates $p < 0.05$ as assessed by Mann-Whitney-U-test.

	**Normal Range**	**Partial LD**	**Generalized LD**	***p***
n		55	5	
LRG1 (ng/l)		17.3 (9.8)	25.5 (40.9)	0.307
Age (years)		43 (22)	24 (31)	0.091
Gender (male/female)		10/45	2/3	-
BMI (kg/m^2)	18.5–24.9	25.3 (4.1)	18.8 (4.9)	<0.001 *
WHR	F: <0.85; M: <0.90	0.97 (0.11)	0.98 (0.10)	0.983
SBP (mmHg)	<140	130 (18)	133 (50)	0.831
DBP (mmHg)	<90	81 (12)	75 (39)	0.851
HbA1c (%)	<6.5%	6.0 (2.0)	5.6 (3.6)	0.626
HbA1c (mmol/mol)	<48	42.4 (22.5)	37.8 (39.4)	0.599
FG (mmol/l)	3.9–5.6	5.5 (3.7)	6.0 (10.2)	0.741
FI (pmol/l)	20–144	112.1 (99.1)	138.8 (231.8)	0.584
HOMA-IR	<2.0	4.9 (5.7)	7.4 (18.1)	0.511
Cholesterol (mmol/l)	<5.20	5.29 (1.94)	5.09 (4.67)	0.800
HDL cholesterol (mmol/l)	>1.03	0.92 (0.54)	0.54 (0.39)	0.003 *
LDL cholesterol (mmol/l)		2.92 (1.77)	0.69 (1.52)	0.006 *
TG (mmol/l)	<1.70	2.83 (3.24)	10.95 (18.25)	0.023 *
FFA (mmol/l)	0.10–0.45	0.61 (0.28)	0.59 (0.40)	0.974
Creatinine (µmol/l)	45–84	68 (23)	37 (24)	0.001 *
eGFR (ml/min/1.73m^2)	>90	99.6 (25.0)	146.3 (72.3)	0.008 *
CRP (mg/l)	<5	1.6 (2.4)	7.6 (7.9)	0.022 *
Adiponectin (mg/l)		2.9 (4.1)	0.2 (1.3)	<0.001 *
Leptin (µg/l)		4.7 (4.4)	0.2 (1.3)	<0.001 *
ALAT (µkat/l)	0.17–0.58	0.47 (0.35)	1.01 (0.86)	0.012 *
ASAT (µkat/l)	0.17–0.6	0.47 (0.27)	0.95 (0.69)	0.002 *
GGT (µkat/l)	0.1–0.7	0.63 (0.47)	1.19 (5.86)	0.385
